# Effects of Harvest on the Sustainability and Leaf Productivity of Populations of Two Palm Species in Maya Homegardens

**DOI:** 10.1371/journal.pone.0120666

**Published:** 2015-03-24

**Authors:** Andrea Martínez-Ballesté, Carlos Martorell

**Affiliations:** 1 Jardín Botánico, Instituto de Biología, Universidad Nacional Autónoma de México, México, D.F. Mexico; 2 Departamento de Ecología y Recursos Naturales, Facultad de Ciencias, Universidad Nacional Autónoma de México, México, D.F. Mexico; Tennessee State University, UNITED STATES

## Abstract

Traditional management practices are usually thought to be sustainable. The Maya manage *Sabal* (Arecaceae) palms in homegardens, using their leaves for thatching. The sustainability of such production systems depends on the long-term persistence of palm populations, whereas resource availability also depends on the number of leaves on individual palms. We examined how leaf harvest affects *Sabal yapa* and *S*. *mexicana* population growth rates (λ) and leaf production, comparing traditional and alternative harvest regimes in terms of sustainability and productivity. Demographic, harvest and leaf production data were recorded for three years in two homegardens. We used general integral projection models linked to leaf-production models to describe population dynamics and productivity. Harvest had no effect on *S*. *yapa*’s vital rates or on λ, but it changed the growth rate of individuals of *S*. *mexicana*, with a negligible impact on λ. Homegardens affected λ values, reflecting the species’ ecological affinities. *S*. *mexicana*, introduced from mesic forests, required watering and shade; therefore, its population declined rapidly in the homegarden that lacked both water and shade. The λ of the xerophilic *S*. *yapa* was slightly larger without watering than with watering. Palms usually compensated for leaf extraction, causing the number of leaves harvested per individual to increase with harvest intensity. Nevertheless, traditional management is relatively mild, allowing standing leaves to accumulate but reducing the homegarden’s yield. Apparently, the Maya do not seek to maximize annual production but to ensure the availability of large numbers of leaves in homegardens. These leaves may then be used when the entire roof of a hut needs to be replaced every few years.

## Introduction

Non-timber forest products provide resources to numerous families from rural communities. Due to the importance of these resources, people have developed diverse procedures for their management. These procedures range from low-intensity gathering in natural vegetation to the incorporation of plant resources into agroforestry systems, in which management is more intense and attempts to ensure the long-term availability of non-timber forest products.

It has been assumed that the impact of management on the population dynamics of a plant species is in part determined by the plant part harvested (i.e., flowers, fruits, branches, boles), the harvesting frequency [1], and the form and intensity with which populations are managed. Agroforestry systems are constructed spaces in which farmers attempt to emulate certain degrees of structural complexity and biodiversity [2]. In agroforestry systems, decisions about management are made in an integrated manner; therefore, practices favoring certain resources may either be positive or negative for other coexisting resources.

Palms produce widely used non-timber forest products worldwide [3,4] and are mostly used for the extraction of leaves, trunks, and fruits. The economic and cultural importance of palms has generated interest in understanding the impact of extraction on population dynamics to allow the sustainability of management practices to be evaluated. Studies testing various defoliation treatments in palm species such as *Chamaedorea tepejilote*, *Chamaedorea radicalis*, and *Sabal uresana* have revealed the effects of extraction on the vital rates of palm populations, i.e., growth, survival, and fruit production [5–7].

Understanding vital rates is important for two reasons: first, they determine the population growth rate and thus whether the population will endure by itself or if additional management practices such as planting are required. Second, vital rates shape population structure (i.e., the proportion of individuals with different sizes). Given that palm size governs the production of new leaves [8,9], population structure determines the amount of resource available for harvest. Hence, the productivity and sustainability of the system depend on processes occurring at two different levels of organization: the population, whose persistence depends on its growth rate, and the individual, which produces the leaves. Both levels need to be accounted for to fully understand the dynamics of the production system.

Palms from the genus *Sabal* have been managed by Mayan people in the Yucatan peninsula (Mexico) since Pre-Columbian times, and their populations have been introduced in homegardens for the extraction of leaves used as thatching material for houses [10]. Maya people need a continuous supply of leaves for repairing the roofs of their homes. Also, every 15 years, roofs need to be renewed completely [11], and occasionally new buildings are constructed. Such events require large amounts of leaves that are mostly produced in the homegarden. Maya also sell leaves. Although this is still infrequent, it may eventually become an important source of income given the increasing demand of the resource [11]. Traditional harvest methods may change in the future to meet this expanding market.

In the present work, we describe the results of a three-year study of populations of two palms, *Sabal yapa* Wright ex Becc. and *Sabal mexicana* Mart., which grow in Maya homegardens and are managed for leaf extraction. We examined how harvesting of *Sabal* palms affects the availability of the resource and the potential for the long-term subsistence of managed populations. In particular, we asked the following questions: How does harvesting affect vital rates, and thus population structure and growth rates? How many leaves are produced by different-sized palms and which is the effect of harvest on leaf production? Finally, how does harvesting affect the overall leaf productivity of the homegarden? To answer these questions, we integrated demographic and leaf-production models to compare the impacts of various management methods on population growth rates and on the amount of resources available in the population.

## Methods

### Study species and site

*Sabal* is a genus of medium to tall single-stemmed hermaphroditic palms growing to between 15 and 20 m in height. The leaves are flabellate and costapalmate, with 15 to 20 segments. Their flowers are arranged in nearly 3-m long inflorescences produced annually between January and March [12]. *Sabal yapa* is the more broadly distributed species on the Yucatan Peninsula, as it is common in seasonally dry tropical forests and secondary vegetation of central and northern Yucatan as well as in grasslands, milpas, and homegardens [10]. *Sabal mexicana* is a broadly distributed species in evergreen to semi-evergreen tropical forests in Mexico; on the Yucatan Peninsula, however, *S*. *mexicana* is only found growing in cultivation in homegardens [12].

Homegardens are located adjacent to houses and are composed of a mixture of species, including primarily fruit trees but also medicinal and ornamental plants and cultivated tubers. This species mixture forms a complex, multi-stratified agroforestry system [13] with high ecological complexity that has been compared to that of rainforests [14].

The Maya consider that an adequate size of palm leaves for harvesting is reached when individuals have formed an evident stem, a condition requiring seven years of growth during which leaf anatomy goes through several stages. Once the stem is formed, palms up to 3 m in height are harvested. Leaves from taller individuals are harvested only occasionally due to the risk involved in climbing stems that may reach up to 11 m. Harvesting is conducted once or twice each year by cutting the older leaves first, leaving one to two leaves besides the apical sprout. Leaves that wilt on the palm are cut and discarded because they are not useful for thatching. Palms are never cut down for harvesting.

The demographic study was made in two homegardens, designated H1 and H2, in the village of Maxcanú, Yucatan, Mexico. The gardens had surface areas of 0.61 and 0.25 ha, respectively. In both homegardens, species of the genus *Sabal* are grown together with other tree and shrub species used for several purposes. H1 presented a high density of cultivated trees, had irrigation, and received weeding practices. In H2 the tree cover was smaller, and seeds of *S*. *yapa* were sown every several years and protected from domestic animals.

### Ethics Statement

Before beginning our fieldwork, we orally asked for authorization from Maria Belem and Librada Kumul, the owners of the privately owned homegardens in which our research was conducted. For future permissions, either they or their successors should be contacted. We informed the Municipal Presidency of Maxcanú about the research we intended to conduct and received their oral authorization in response. Because we conducted in situ sampling without gathering vouchers of populations of *Sabal mexicana* and *S*. *yapa*, we did not apply for a SEMARNAT-08-049 collection permit from the Mexican Federal Environmental Agency (SEMARNAT) because no specific permissions were required for these locations/activities. The two species sampled in the field studies were not endangered or protected species under the Mexican official norm NOM-059 SEMARNAT. The specific location of our study was at coordinates 20° 35' 00" N 90° 00' 30" W and 20° 34' 50" N 89° 59' 50" W, respectively, for H1 and H2.

#### Data collection

Within both homegardens, palms of *S*. *yapa* and *S*. *mexicana* were sampled in randomly chosen 20-m long transects whose width depended on the size of the palm (1 m for seedlings, 6 m for palms < 3 m in height, and 12 m for larger individuals). 811 and 809 individuals of *S*. *yapa* and *S*. *mexicana* respectively were sampled. Between 1998 and 2000, the survival and annual growth of individuals from both palm species were recorded. In all studied years, the recruitment of new individuals and the number of fruits produced were recorded (see [15] for further details). To record fruit production, we climbed the palms with stems larger than 3 m. Because palms in general do not form a seed bank [16,17], it was assumed that all produced seeds would die if they did not germinate. For this reason, the seed category was excluded from the demographic model.

To estimate leaf production and harvest, we marked the youngest leaf and counted the number of leaves present on each individual every year. This allowed us to estimate the number of new leaves produced annually because they are formed above the marked leaf. Palms were freely selected for extraction by the householders during the entire study. Thus, we also estimated the number of leaves harvested as the difference between the number of leaves on the palm at the beginning of each year and the number of old leaves left at the end of the year. We recorded if any leaves had wilted and thus were unusable for thatching. Such leaves were yellow and evidently dry. We were occasionally asked to harvest some leaves from the individuals we climbed to measure fecundity. This allowed us to estimate the effects of harvesting in large palms, but these data were excluded from the analyses of traditional harvest.

### Demographic and leaf production models

Structured population models are based on the premise that the individuals in a population behave differently according to their attributes. Among the attributes, age and size are the most common descriptors of the status of individuals. In modular organisms, size is usually highly correlated with demographic processes such as survival, growth and reproduction [18]. In general, palm size can be accurately measured as stem length. Traditionally, matrix models that require splitting length into discrete size categories are used to analyze the demographic behavior of populations of palms and other plant species. Categorization may bias population growth and structure estimates, a problem that is solved by the use of integral populations models (IPMs) that recognize the continuous nature of size variables. IPMs also require smaller samples and make it easier to integrate covariates such as harvest intensity into the analyses [19–21].

Young *Sabal* individuals lack an evident stem for many years. Important changes in the individual take place during this period, encompassing various plant stages ranging from seedlings to large palms that are being already harvested. Such stages were measured in a discrete scale, but stem length was used to describe stemmed palms. Thus, we used a general integral projection model (GIPM) that allowed us to model populations that are structured by both discrete developmental categories and continuous variables [22]. To do so we recognized two domains: **D**, corresponding to individuals without the aboveground stem, which were categorized into five groups (Table 1); and **Ω**, in which plant behavior is described as a function of stem height. Based on field data, we assumed **Ω** to run from 0 to 9 m. Leaf harvest starts in I3 individuals and may continue along the whole **Ω** domain.

**Table 1:** Criteria for classifying plants in the D domain based on leaf morphology.

Category	Definition	*S*. *yapa* (*n* = 811)	*S*. *mexicana* (*n* = 809)
S1	A single undivided leaf	100	141
S2	Two or more undivided leaves	345	374
I1	Leaf divided in two portions	111	81
I2	Incompletely divided palmate leaf	110	106
I3	Completely divided palmate leaf	145	108

Plants in this domain do not have a developed aboveground stem.

The GIPM summarizes the behavior of individuals using the complex function *K*(*x*,*y*), known as the kernel. The time is assumed to be discrete. If *n*(*x*,*t*) is a function describing the population structure at time *t*, then

n(y,t+1)=∫K(x,y)n(x,t)dx, (1)

where *x* is the stage or size of an individual at time *t*, and *y* is its stage or size at time *t* + 1. The kernel, in turn, can be decomposed into a set of functions that correspond roughly to the different vital rates: survival, growth and reproduction. Using generalized linear models we assessed whether these functions differed significantly among homegardens and years. The effect of harvesting was assessed by testing whether the proportion of leaves harvested per individual had a significant effect on its vital rates. Only factors (homegarden, harvest intensity, year, and size) having a significant effect on vital rates were included in the GIPM (see S1 Appendix for kernel structure and model fitting procedures); therefore, the differences in the estimated population growth rates and structure cannot be attributed to chance.

The number of leaves available on an individual at time *t* + 1, *L*_*t+*1_, equals the number of leaves it had minus those harvested, *L*_*h*,*t*_ plus those newly produced, *L*_*p*,*t*_. However, if leaves are not harvested, they wilt and become unsuitable for thatching. We observed that this occurred in individuals that had not been harvested for a long period; therefore, it appears that there is a maximum number of leaves *L*_max_ that an individual may have. Thus, we used the formula

$$L_{t+1}=min\left(L_{t}-L_{h,t}+L_{p,t},L_{max}\right).$$ (2)

In this way, only usable leaves were included, whereas wilted ones were discarded. As before, we tested whether *L*_*p*,*t*_ changed depending on the homegarden, year, harvest intensity, and plant size using generalized additive models because the function relating leaf production to size seemed highly non-linear. The observed harvest intensity *L*_*h*,*t*_ depending on the homegarden, year, and plant size was also estimated also using generalized additive models. To estimate *L*_max_ we used stochastic frontier regressions. Unlike ordinary regression, this analysis fits a line that determines the maximum (not the mean) number of leaves that a palm may have depending on its size (see S1 Appendix for details on the statistical procedures).

### Simulations

We simulated population dynamics and leaf production for 21 different harvesting regimes: the traditional regime_,_ consisting of annually extracting the observed proportion of harvested leaves; five harvesting regimes differing in the proportion (0, 0.2, 0.4, 0.6 or 1) of leaves being harvested; and five regimes in which different numbers (1, 2, 3, 4 or 5) of leaves were left unharvested on each individual. The latter were selected because the Maya traditionally base their management on the number of leaves left on the palm rather than on a proportion of harvested leaves. The ten alternative regimes had two variants: one in which leaves were cut from all harvestable individuals (I3 plus all stemmed plants) and another in which only I3 plus individuals with stems < 3 m were cut. The latter regime roughly corresponds to that commonly followed by the Maya. In each iteration of the model, we estimated the number of harvested leaves according to one of the harvest regimes, and we used equation 2 to calculate the number of leaves available for the next iteration’s harvest.

The dynamics of the population was simulated from equation 1. Because the vital rates and thus the kernel may depend on the harvest, i.e., the proportion of leaves harvested, we calculated this figure from the number of available leaves and the management regime, and we estimated a new kernel for every iteration of equation 1. Temporal variability was introduced in the model by means of a stochastic GIPM [20], randomly selecting the kernel parameters for one of the three years in each iteration. To estimate population growth and structure, we iterated the model 5,000 times and discarded the first 500 results to allow the system to reach its stationary size structure (SSS) [22]. We calculated the annual *λ*-values as

$$\lambda =\frac{\int n\left(x,t+1\right)dx}{\int n\left(x,t\right)dx},$$ (3)

and the stochastic population growth rate, *λ*_*s*_, as the geometric mean of the annual *λ* values [22]. The population size structure at time *t* was standardized to one and averaged across the last 4,500 iterations to estimate the mean of the SSS (see S1 Appendix for details).

We used *λ*_*s*_ as a measure of the sustainability of the different harvest regimes. If *λ*_*s*_ ≥ 1, the population size is expected to remain unchanged or to increase in the long term, indicating that the resource is not expected to fail in the future. Leaf production was estimated for each regime as the mean number of leaves collected annually per harvestable individual, *H*_*t*_, as

$$H_{t}=\frac{\int_{D}n\left(x,t\right)L_{h}\left(x,t\right)dx}{\int_{D}n\left(x,t\right)dx}+p\left(\text{I3},t\right)L_{h}\left(\text{I3},t\right),$$

where *L*_*h*_(*x*,*t*) is the number of leaves harvested from size-*x* palms and *p*(I3,*t*) and *L*_*h*_(I3,*t*) are the fraction of individuals and the number of leaves harvested in category I3, respectively. Averaging *L*_*p*,*t*_, *L*_*h*,*t*_ and *H*_*t*_ over the last 4,500 iterations, we estimated the mean number of leaves available and harvested annually from different-sized palms and the mean per capita leaf harvest. Because I3 and stemmed individuals have large crowns and thus require a considerable area to grow, *H*_*t*_ indirectly provides a measure of productivity per unit of area of the homegarden devoted to growing palms.

## Results

### Effects of harvest on vital rates

In this section, we only include the results relevant for understanding population dynamics and structure. Because individuals without a stem are not harvested and contribute relatively little to population growth rates [15], we focus on plants with evident stems. The detailed results of the statistical analyses of vital rates and of stemless individuals are reported in the S1 Appendix.

**Fig 1:**
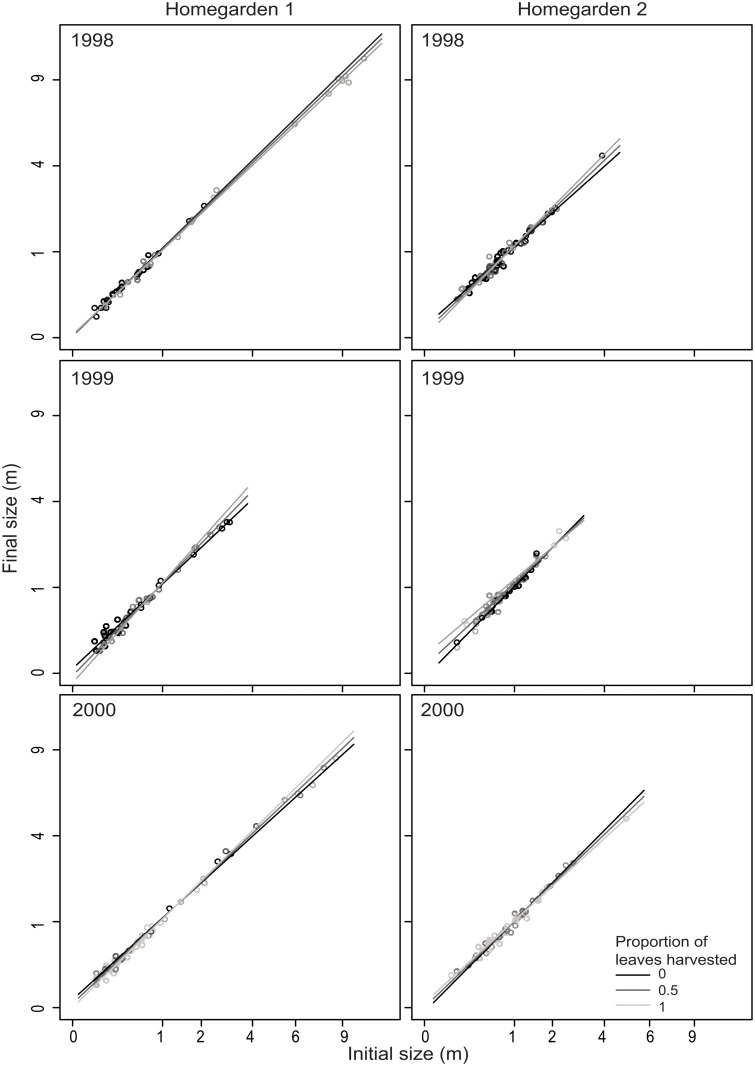
Growth of *Sabal mexicana* stems depending on the proportion of harvested leaves in homegarden 1 (left) and homegarden 2 (right). Axes’ scale is square root transformed.

The survival of *S*. *mexicana* was higher in H1 than in H2, but the opposite occurred for *S*. *yapa* (binomial regression *P* ≤ 0.033). Fruit production of both species was greater in H2 than in H1 (Poisson regression *P* ≤ 0.003, Fig. A2 in S1 Appendix). The growth of *S*. *yapa* differed between homegardens and between years (normal regression *P* ≤ 0.006). The only vital rate affected by harvest intensity was the growth rate of *S*. *mexicana*. The effects of harvest intensity on growth behaved idiosyncratically depending on height, year, and homegarden (binomial regression *P* < 0.001, Fig. 1): in H1, harvesting reduced the growth of palms less than 1 m tall but accelerated the growth of taller individuals; inversely, harvesting in H2 accelerated the growth of smaller individuals but decreased the growth rate of those taller than 2 m (Fig. 1).

### Population growth and structure

Because *S*. *yapa*’s vital rates were unaffected by harvest, the population growth and size structure of this species remained constant in all harvest regimes. In this species, population growth was close to equilibrium, but the number of palms tended to decline in H1 (*λ*_*s*_ = 0.985) and to increase in H2 (*λ*_*s*_ = 1.023). At the stationary size structure, the number of individuals showed the typical inverse-J size distribution (result not shown).

**Fig 2:**
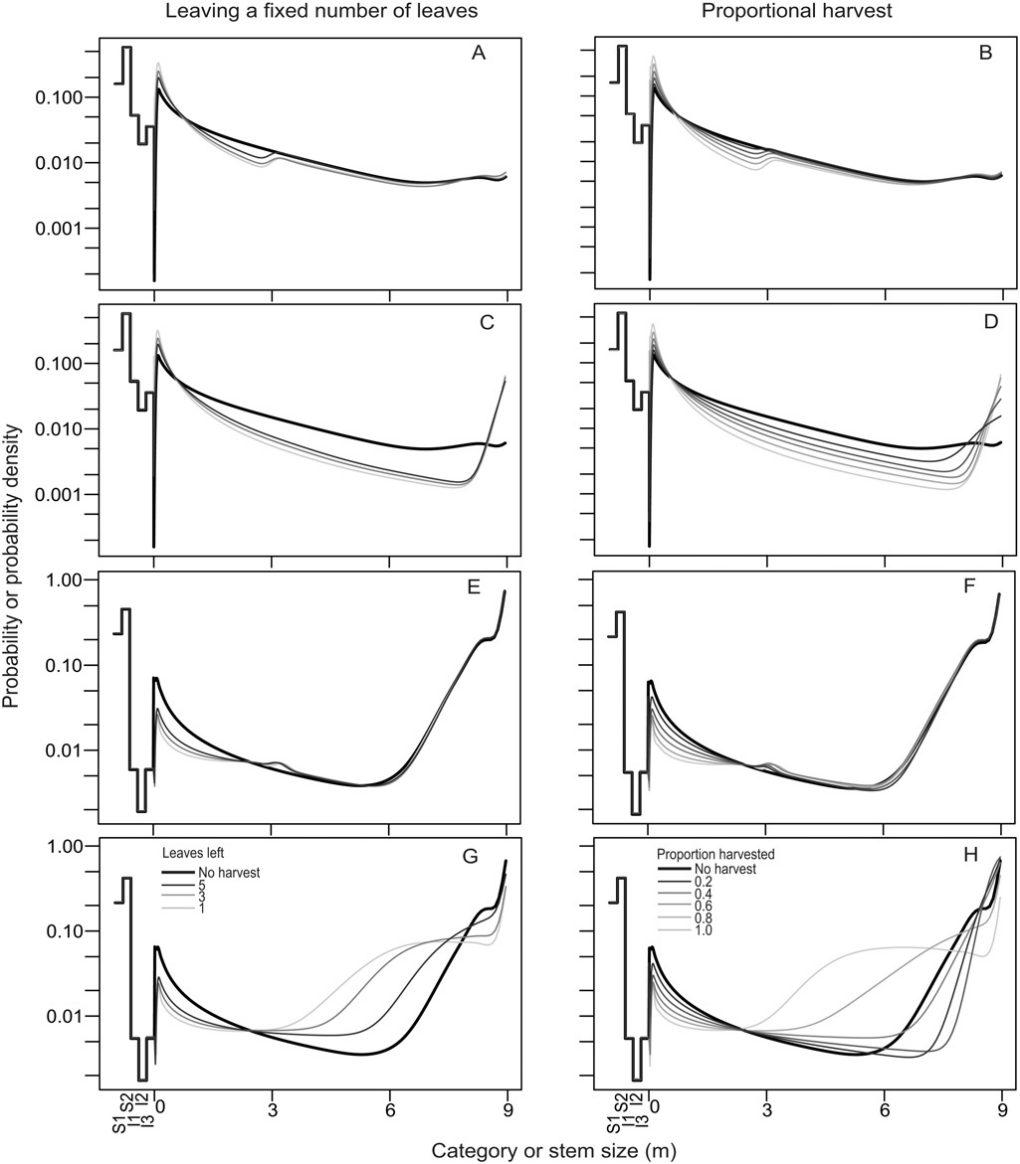
Population stationary size structures of *Sabal mexicana* under different harvest procedures: leaving a fixed number of leaves on the palm (left) and harvesting a constant proportion of leaves (right). Lighter colors correspond to more intense harvesting. In all cases, I3 individuals were harvested, together with either all stemmed palms (all) or only those measuring less than 3 m (<3 m). A-B: homegarden 1, <3 m. C-D: homegarden 1, all. E-F: homegarden 2, <3 m. G-H: homegarden 2, all.

The population growth rates of *S*. *mexicana* showed the opposite pattern to *S*. *yapa*, with populations increasing by approximately 1% annually in H1 but rapidly declining in H2 (> 6% annually). Leaf harvest caused only a negligible (< 0.002) reduction in *λ*_*s*_. As in *S*. *yapa*, the stationary size structure of *S*. *mexicana* in H1 displayed a progressive reduction in the number of individuals as their corresponding class category or stem size increased (Fig. 2 A-D). The stationary size structure in H2 differed in that individuals over 6 m tall were more abundant than in H1 (Fig. 2 E-H). The effects of harvest on the stationary size structure reflected changes in the growth rates of individuals due to harvesting. Thus, as harvest intensity increased, the frequency of 1-m individuals in H1 increased at the expense of the frequency of the taller classes (Fig. 2 C and D), whereas in H2, individuals shorter than 2 m became rarer as higher palms became more abundant (Fig. 2 G and H). In H2, as harvest intensity increased, the number of 3- to 8-m tall individuals also increased, although the tallest individuals (> 8 m) decreased in number relative to unharvested populations (Fig. 2 G and H). Not harvesting palms over 3 m in height had no effect on the frequency of taller individuals (Fig. 2 A, B, E, and F).

### Leaf harvest and production

Our data confirmed the information provided by householders that plants taller than 3 m were rarely harvested. In general, palms with 1.5- to 3-m tall stems were most intensely harvested (generalized additive model, *P* < 0.001; see Fig. A3 in the S1 Appendix).

**Fig 3:**
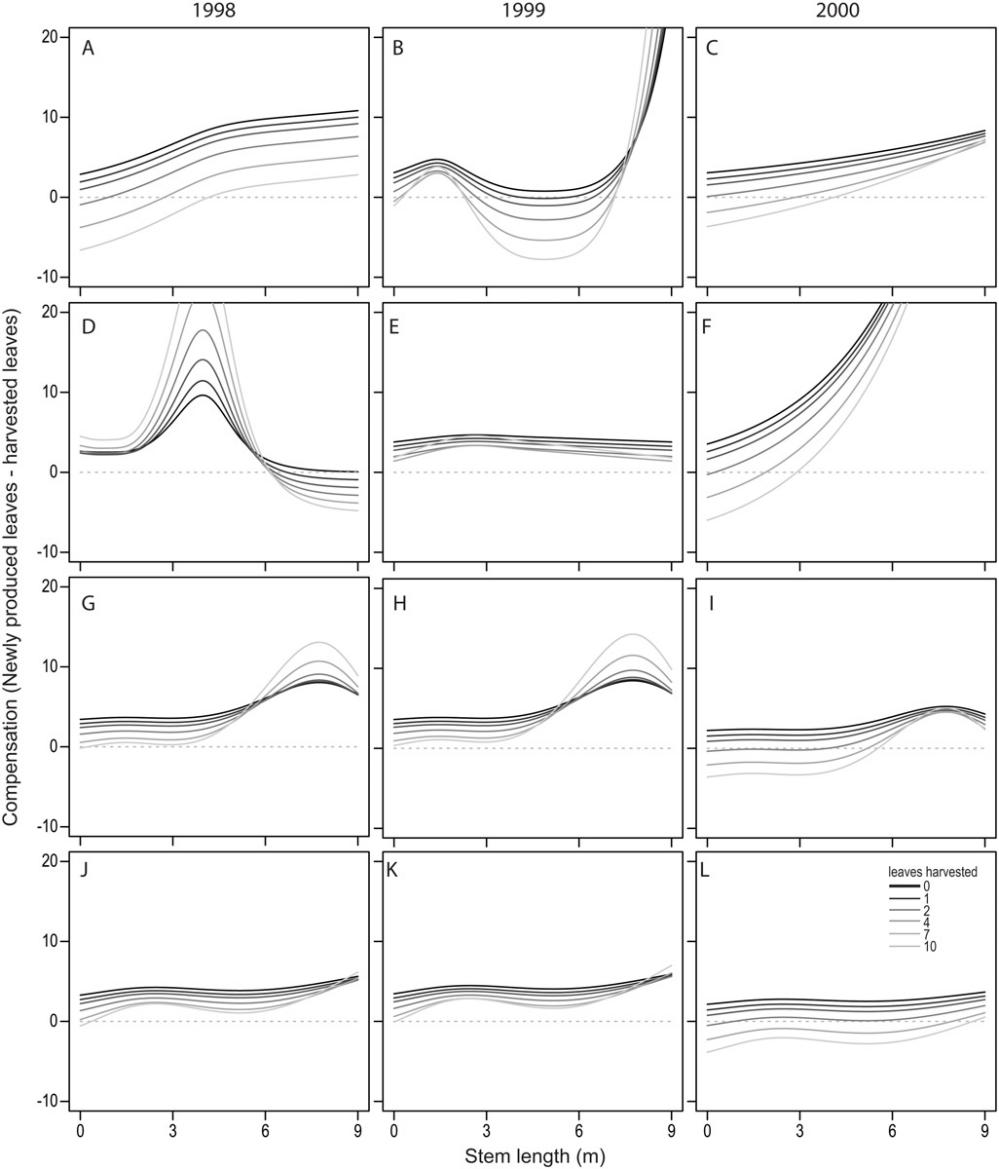
Compensation by different-sized plants of *Sabal yapa* and *S*. *mexicana* in different years and homegardens. A-C: *S*. *yapa*, homegarden 1; D-F: *S*. *yapa*, homegarden 2; G-I: *S*. *mexicana*, homegarden 1; J-L: *S*. *mexicana*, homegarden 2.

In general, leaf production increased with size and as the harvest became more intense (Poisson regression *P* < 0.001). The production of new leaves also differed between homegardens (*P* < 0.002) and between years (*P* < 0.012). Estimating compensation as the number of leaves produced minus the number of leaves harvested, we found that, in most cases, palms were capable of compensating for harvesting (compensation > 0). However, this was not true in all years and homegardens (Fig. 3). The capacity for compensation varied between years, apparently due to precipitation. In the dry years 1998 and 2000 some palms—particularly the smaller ones—were incapable of compensating for the losses due to harvest (Fig. 3).

**Fig 4:**
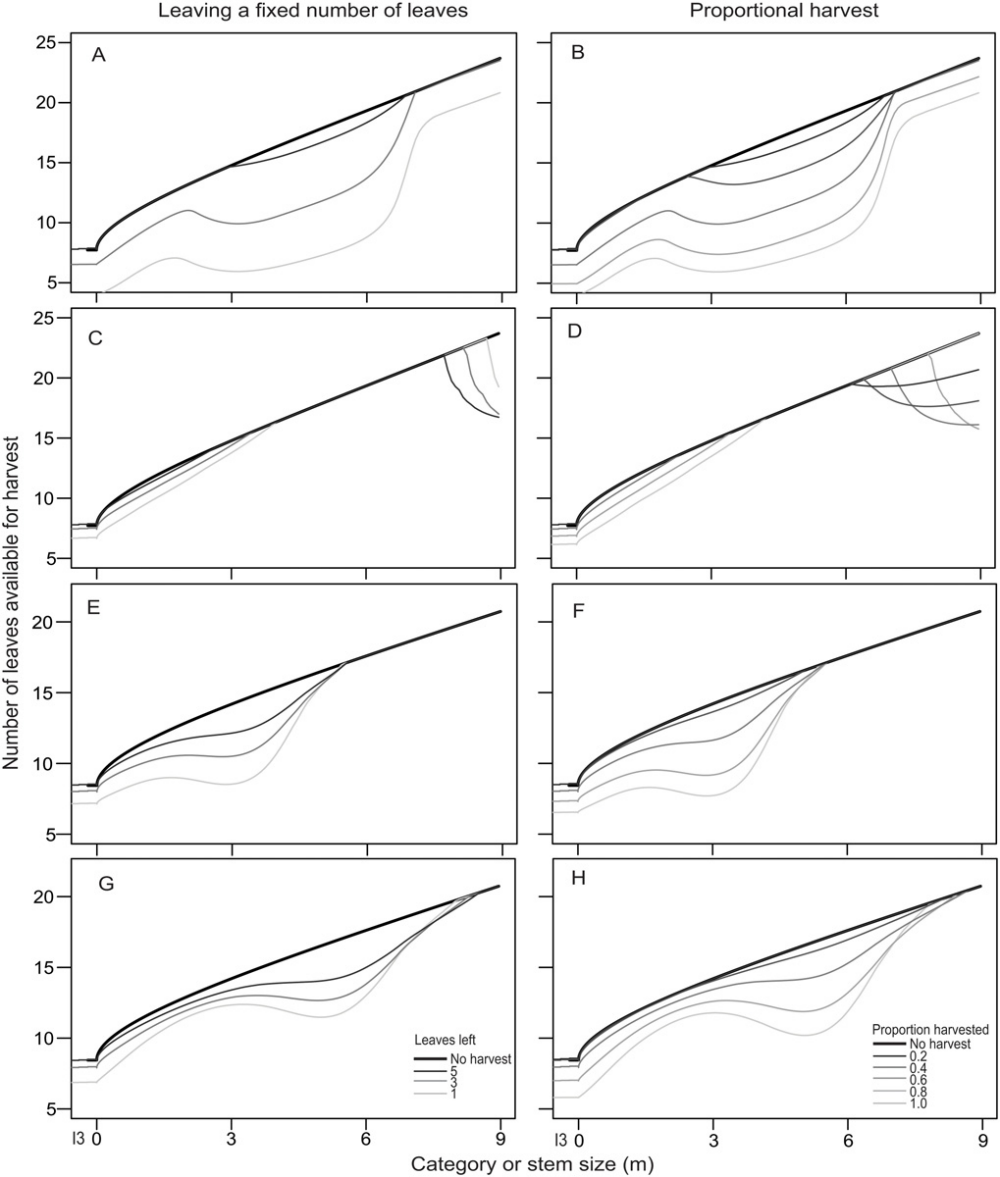
Number of leaves available in plants of different sizes under different harvest regimes. Two different harvest methods are shown: leaving a fixed number of leaves on the palm (left) and harvesting a constant proportion of leaves (right). Lighter colors correspond to more intense harvesting. In the simulations, I3 and all stemmed palms were harvested. A-B: *S*. *yapa*, homegarden 1. C-D: *S*. *yapa*, homegarden 2. E-F: *S*. *mexicana*, homegarden 1. G-H: *S*. *mexicana*, homegarden 2.

For both species, the maximum number of leaves that can be on a palm before they start wilting (*L*_max_) increased with size from approximately 7 leaves on smaller individuals to slightly over 20 on the largest ones (stochastic frontier regression *P* < 0.001). Taller plants also tended to have more leaves available for harvest in both species and homegardens regardless of the harvest regime (Fig. 4). In all cases, unharvested individuals reached *L*_max_, and the number of available leaves diminished as the harvest intensified. In general, larger individuals reached *L*_max_ in at least mild harvest regimes, with the exception of very large (>7.5 m) *S*. *yapa* in H2 (Fig. 4 C and D).

**Fig 5:**
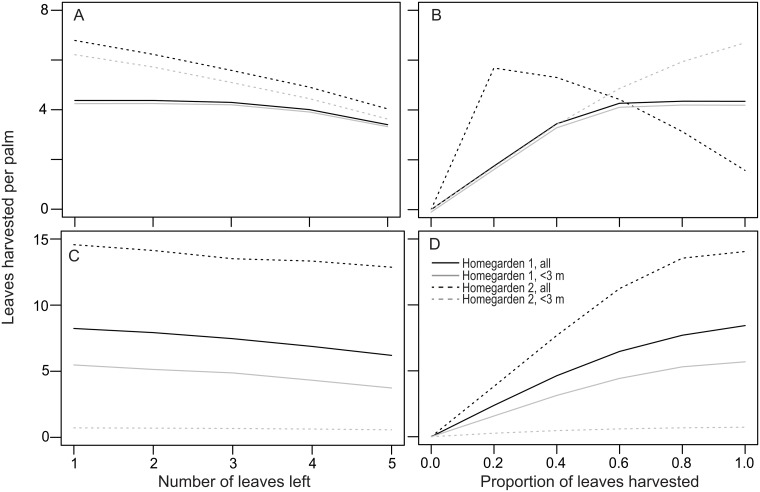
Leaf production for different harvest intensities in both homegardens. Two different harvest procedures are shown: leaving a fixed number of leaves on the palm (left) and harvesting a constant proportion of leaves (right). For each method, simulations were performed harvesting either all plants with stems (all) or only those measuring less than 3 m (<3 m). I3 individuals were always harvested. A-B: Per capita number of leaves harvested annually from *S*. *yapa* individuals. C-D: The same as A-B but for *S*. *mexicana*.

In both species and homegardens, the average number of harvested leaves per palm in the population was greater when all individuals were harvested than when only individuals smaller than 3 m were harvested (a scenario in which zero leaves are harvested from many individuals). In general, the yield was greater when the harvest intensity increased. Such a trend was less apparent under the harvesting regimes using only palms with stems shorter than 3 m (Fig. 5). The differences in yield were also more evident in populations of *S*. *mexicana* in H2 (Fig. 5 C and D). Note that in H2, the number of leaves harvested from *S*. *yapa* was highest when the proportion of leaves harvested was approximately 0.2–0.4 if all individuals were harvested, followed by a rapid decline in the yield to less than one leaf per palm when all leaves were harvested annually. In contrast, leaf harvest per palm was negligible in H2 when only < 3 m tall individuals were harvested (Fig. 5 B). In the simulations in which we applied the observed harvest regimes, *S*. *yapa* produced 1.12 and 1.37 leaves per capita in H1 and H2, respectively. For *S*. *mexicana*, the corresponding figures were 2.45 and 0.84. These numbers are much lower than the maximal yields projected using the alternative harvest regimes (Fig. 5).

## Discussion

Our results show that differences in population growth rates, size structures, and rates of production of new leaves by *Sabal* are related to management practices in each homegarden. The behavior of each species appears to be dependent on its biological characteristics, the microenvironmental conditions in homegardens, and, for *S*. *mexicana*, harvest intensity. Leaf production increased concomitantly with harvest intensity, and usually compensated leaf extraction, so that populations were able to tolerate regimes with more intense harvesting rates than those in the traditional harvesting regime.

### Effects of management on vital rates and population dynamics

Significant effects of harvest on vital rates of palms have been described in the literature [23–27]. However, in general, harvesting had no effect on the vital rates of either of the *Sabal* species we studied. The reason for this finding may be that our study was too brief to detect the cumulative effects of harvesting on individual palms. Other studies have reported long-term effects of continued defoliation on population vital rates, which may become apparent only after six years of defoliation [25,27]. In fact, the production of *Sabal* leaves did not differ between harvesting regimes until the second year of their application in an experimental study [28].

The only vital rate that responded to defoliation was the growth rate of *S*. *mexicana*. The production of new leaves involves new internodes and, hence, the elongation of stems [6,29–32]. Consequently, based on our results showing that *Sabal* increased its leaf production as harvesting was intensified, leaf harvesting should have had positive effects on growth. However, more rapid growth of harvested palms was not always observed (for experimental evidence on the lack of correlation between leaf production and stem growth in *Sabal*, see [28]. Only in H2 (in which solar radiation in the understory was higher than in H1) did harvesting stimulate the growth of small palms. This finding agrees with studies stating that only when solar radiation is high does the photosynthetic capability and relative growth rate of palms respond positively to defoliation [8,33].

The effect of harvesting on the individual growth of *S*. *mexicana* palms caused changes in the population’s stationary size structure. Harvest caused the frequency of individuals having certain stem heights to increase as a result of a reduction in their growth rate. This may be because, given that their height does not appreciably change with time, they tend to accumulate in the population. We observed the opposite trend in plants whose growth was stimulated by harvesting. As a result, in H2 there was an increase with harvest in the abundance of individuals > 2 m in height, whereas in H1 the frequency of shorter (< 1 m) individuals increased.

Such changes in the size structure of the palm population may be driving the disappearance of *Sabal* from homegardens. In shaded systems, harvest would increase the availability of small individuals that the Maya prefer for harvesting given their size. However, when the householders abandon their traditional practices, the density and diversity of tree species in the homegardens diminishes [2,13]. Under such high-irradiance conditions, our model predicts a reduction in the proportion of 1–3 m tall individuals, and a high frequency of tall palms. This projection agrees with the observation that very large palms dominate homegardens owned by westernized householders [34]. Under such conditions, leaf production may become extremely low or difficult. As a result, the owners fell the remaining palms, leading to the disappearance of the resource from the household, as it has been observed [34].

In contrast, the differences in population growth rates between both homegardens were not to attributable to harvest regimes, which had a negligible effect on *λ*_*s*_, but to environmental conditions resulting from the homegarden management. Although climate in both homegardens is identical, edaphic humidity appeared to be higher in H1 than in H2 due to irrigation and deeper shade. These differences may explain the higher survival rate (the vital rate with the largest impact on the population growth of *Sabal* and other palms [6,15,24,26]) of *S*. *mexicana* in H1 and of *S*. *yapa* in H2, given that the conditions in these two homegardens resemble those in which both species grow naturally: *S*. *mexicana* grows in humid, shaded understories of evergreen and semi-evergreen tropical forests in Mexico, whereas *S*. *yapa* is native to the drier, more illuminated tropical forests of the Yucatan Peninsula [12,35].

Hence, the appropriate management of microenvironmental conditions in a homegarden apparently determines the long-term persistence of *Sabal* populations. Our results show that Maya farmers could increase the harvest intensity without negative consequences for population-level sustainability, but appropriate management of shade and irrigation are crucial: without substantial water inputs, *S*. *mexicana* was unable to maintain a viable population. Importantly, it must be recognized that numerous plant species are managed in homegardens and that practices can be differentially beneficial for each species. In our case, the management of irrigation and shade is designed based on the functioning of the whole plant community in the productive system, so changing the management regime to only favor one of the *Sabal* species would have detrimental impacts on other species.

### Leaf production

With few exceptions, palms are highly tolerant to defoliation, and leaf production increases with harvesting intensity [5,25,32,36]. As a result, palms are able to compensate for the number of leaves extracted, although that capacity is reduced under low light intensity [8,17,33]. Both species of *Sabal* fit this general pattern: leaf production increased with harvest intensity, in most cases even overcompensating for the loss of leaves. Additionally, compensation was in general higher in H2, where light availability was higher relative to H1. In certain palms, the overcompensation is achieved through the mobilization of stored resources, which are more abundant in larger organisms [8,9]. This may explain why taller individuals of both *Sabal* species could, in general, better compensate for the impacts of harvesting.

The availability of leaves in palms with different stem lengths depended on the capacity of individuals to compensate for harvest and on the maximum number of leaves that a palm could hold before they wilt. An increase in harvesting intensity prevented palms from reaching the maximum number of leaves that would be observed if the palms were left unharvested. This only occurred in palms with stem heights that could not fully compensate for harvesting, even if this occurred in only one year. For example, *S*. *mexicana* in H1 always overcompensated for harvesting except for individuals under 5 m in height in the driest year (2000). As a consequence, a reduction of up to 50% in available leaves was observed (see Fig. 4 E-F, 3-m tall individuals). This result emphasizes the importance of dry years in the availability of the resource, as also observed in other palm species [17,37]. The low intensity of the observed harvest intensity may in general preclude undercompensation in dry years (Fig. 3 I) and thus may respond to the need to have large amounts of leaves available per individual.

An important consideration for the sustainability of the system is to ensure an adequate yield from the homegarden in terms of leaf production. Our results showed that by harvesting only palms under 3 m in height, the Maya obtain fewer leaves per palm than if they harvested all individuals with an apparent stem. This result was more clearly observed in the population of *S*. *mexicana* in H2, in which yields under the traditional harvesting regime were minimal, possibly due to the large proportion of *S*. *mexicana* palms over 3 m tall that occupy a considerable area of the homegarden but render a null contribution to the household’s leaf income.

In nearly all cases, leaf production per capita increased with harvesting without leading to a collapse in leaf production at any point. Even when defoliation reduced the frequency of harvestable (<3 m tall) palms (in H1 the amount of individuals < 1 m in height increased, but they produce relatively few leaves), the capacity of *Sabal* for producing more leaves as harvesting intensified compensated for that effect. Only in *S*. *yapa* in H2 did the yield decrease when over 20% of the leaves were extracted under the regime in which all palms were harvested. This may be due to the observed incapacity of taller plants, which produced more leaves and thus contributed to per capita leaf yield, to compensate for the high harvest intensities. On the contrary, the remarkable increment in yield per capita of *S*. *mexicana* in H2 when all palms were harvested reflects the considerable increase in the abundance of individuals between 3 and 8 m in height, which produce the largest number of leaves.

### The logic of traditional management

Population growth rates close to 1 indicate that the population sizes of both species may undergo only small changes in the future, ensuring the availability of the resource for a relatively long time. In contrast, exploitation of *S*. *mexicana* in H2 is not sustainable, given that in a couple of decades the population would virtually disappear. However, the Maya perform actions that were not considered in our model, such as sowing and seedling protection, which undoubtedly can increase the size of the population if its density decreases to a level below what they consider to be convenient. It is interesting to note that such practices were only recorded in H2, where they may not only maintain a viable population but also augment the number of individuals < 3 m. Without sowing, this homegarden would only have large individuals that are difficult to harvest.

The traditional harvest regime results in relatively low yields. While the method of harvesting all leaves except one or two (which the Mayan farmers say they follow) does result in maximum yields per capita, our data indicate that, in reality, the harvest intensity is much lower, or that many plants are left unharvested. The interruption of harvesting when palms reach over 3 m in height produces an even higher reduction in the productivity of the homegarden.

Therefore, the logic of traditional management does not seem to follow a criterion of maximization of annual yields. The demand for *Sabal* leaves is not constant over time. In general, small amounts of leaves are needed each year for repairing roofs or for minor buildings, such as screens dividing the bathroom from the rest of the homegarden. It has been estimated that a household typically requires 367 leaves per year [11], a number well below the production capacity of the homegarden. Even so, surpluses are rarely sold but are, rather, left to remain on the palms. Every 15 years, the entire roof of a house needs to be totally renewed [11], which requires nearly 1,800 leaves [11]. As shown by our results, if low-intensity harvesting rates are regularly maintained, the palms are capable of keeping crowns with much higher numbers of available leaves than they are under intense harvesting regimes that maximize annual production. Low harvest intensities also allow the palm to compensate extraction even in dry years, and thus may be a form of precluding temporal variability from diminishing leaf availability. All palms over 3 m tall even reached their maximum production capacity in the simulations based on traditional management. The large number of standing palm leaves under traditional management allow farmers to handle the pulses of high demand for leaves associated with roof renovation. In such eventualities, the Maya can hire workers to climb the palms to harvest palms greater than 3 m tall, which function as a sort of savings account.

The abundance and richness of species in homegardens depends on several social, economic and cultural factors. If the production is destined to subsistence, householders usually prefer to produce a large diversity of resources than augmenting the availability of a few; in contrast, commercial production usually results in the opposite [2,13,14]. Thus, the preference for diverse homegardens that satisfy the multiple needs of the household may discourage large-scale commercialization. Nevertheless, our results show that annual per-capita production of leaves can be substantially increased, and thus a greater yield can be attained without increasing the number of palms and compromising the production of other resources in the homegarden. This would come at the cost of reducing the average number of leaves that are available at any given moment, compromising the capacity of the household for self-sufficiency when whole roofs need to be built. Far from responding to the logic of a market economy, the manipulation of conditions and populations in the homegarden has been developed as a management strategy that allows the Maya to face moments of extraordinary resource demand or unusual climatic events, such as drought.

## Supporting Information

S1 Appendix Demographic and leaf-production models. (DOCX)

S1 Dataset Raw data. (XLSX)
