# Supplementary material for: Effects of Harvest on the Sustainability and Leaf Productivity of Populations of Two Palm Species in Maya Homegardens
Source: PLoS One. 2015 Mar 24;10(3):e0120666. doi: 10.1371/journal.pone.0120666 (PMC4372575; doi:10.1371/journal.pone.0120666)
Supplement: S1 Appendix — (DOCX) [file pone.0120666.s001.docx]

**APPENDIX. DEMOGRAPHIC AND LEAF-PRODUCTION MODELS**

**1. Population dynamics**

We used a generalized integral projection model GIPM (Ellner and Rees (2006)) that describes the demographic behavior by means of the function *K*(*x,y*), known as the kernel.

$n\left(y,t+1\right)=\int K(x,y)n(x,t)dx$ (1)

where *x* is the stage or size of an individual at time *t*, and *y* its stage or size at time *t* + 1, and *n* are function describing the number (or probability density) of individuals in the population categorized into developmental stages (**D** domain) or described as having different sizes, a continuous variable (**Ω** domain). Please see the main text for details on how this was done and on general information on GIMPs.

1.1 *Model selection and structure*

Before describing the kernel structure and fitting procedures, a note on notation is required. Variables are represented by Latin characters, whereas Greek ones are used for parameters (constants) estimated from the data. Subindices inside parentheses indicate that a parameter or function depends on the homegarden (*h*) or the year (*k*). Subindices without parentheses are used to distinguish among parameters or functions with similar meanings. All the analyses were performed separately for each species, and only significant terms were included. Thus, the most complex function is shown for each process and some parameters may equal zero in one of the species. A list of all symbols is shown in table A1.

| Table A1. Symbols for the variables, functions, matrices and parameters used in the demographic model | | |
| --- | --- | --- |
| Symbol | Meaning | see eqn. |
| Variables |  |  |
| *h* | Homegarden |  |
| *i* | Category of plant at time *t* + 1 |  |
| *j* | Category of plant at time *t* |  |
| *k* | year (1998, 1998, or 2000) |  |
| *l* | Proportion of leaves harvested |  |
| *t* | Time step |  |
| *x* | Size (or category) of a plant at time t |  |
| *y* | Size (or category) of a plant at time t + 1 |  |
|  |  |  |
| Functions and matrices | |  |
| **A** | Portion of the kernel for transitions within the **D** domain. | 6 |
| *F*(*x*) | Number of fruits produced by a size-*x* individual | 4 |
| *f_i_*(*x*) | Nimber of category *i* seedlings produced by a size-*x* adult | 5 |
| **G** | Growth in the **D** domain. | 7 |
| *g*(*x,y*) | Growth function for the **Ω** domain | 3 |
| *K*(*x,y*) | Complete kernel the for the **D** and **Ω** domains | 1 |
| *k*(*x,y*) | Portion of the kernel for transitions within the **Ω** domain. | 2 |
| *n*(*x,t*) | Population structure at time t | 1 |
| **S** | Survival in the **D** domain | 7 |
| *s*(*x*) | Survival function for the **Ω** domain | 3 |
|  |  |  |
| Parameters |  |  |
| *α_F_, β_F_* | Parameters of the fruit production function | 4 |
| *α_g_, β_g_, γ_g_, δ_g_* | Parameters of the growth function (intercept, and effects of size, harvest and their interaction, respectively) in the **Ω** domain | 3 |
| *γ_i,j_* | Transition (growth) probability of an individual from category *j* to category *i* given that it survives | 7 |
| *γ*_Ω_*_,_*_I3_ | Probability of an I3 individual develops a stem given that it survives | 8 |
| *θ* | Probability that a newly established seedling reaches category S1 | 5 |
| *μ_y_* | Mean length of the stem of plants that have developed it during the previous year | 8 |
| *σ_g_* | Standard deviation of the growth of individuals in the **Ω** domain | 3 |
| *σ_y_* | Standard deviation of the length of the stem of plants that have developed it during the previous year | 8 |
| *ς_j_* | Survival probability for individuals in category *j* of the **D** domain | 7 |
| *ς*_Ω_ | Survival probability for individuals in the **Ω** domain | 3 |
| *φ* | Probability that the seed in a fruit becomes established | 5 |

1.1.1. Transitions within the **Ω** domain

Transitions within this domain were modeled by means of the function

*k*(*x,y*)= *g*(*x,y*) *s*(*x*) (2)

where *g*(*x,y*) is corresponds to growth and *s*(*x*) to survival. Transforming *x* and *y* into their square roots resulted in a linear relationship (*R*^2^ > 0.995) between both variables, and in a nearly normal error (for *S. mexicana*, a Shapiro-Wilk test found no significant deviations from normality in the residuals, and for *S. yapa*, the test was significant [*P* = 0.029], but the statistic *W* = 0.99 was extremely close to 1, the theoretical value expected if data were perfectly normal). Thus, we fitted *g*(*x,y*) using square-root transformed sizes through a normal regression. Using this analysis found that year, homegarden and harvest intensity should be included in the growth model in the case of *S. mexicana*, while for *S. yapa* harvest did not affect growth (Table A2).

Survival was modeled by means of a logistic function of initial size *x*, year, homegarden and leaf harvest (*l*). Neither size (regardless of whether it was included in the model as *x* or √*x*) nor harvest had a significant effect on survival, which was simply a constant that depended on homegarden and year (Table A3). Thus, transitions within the **Ω** domain were modeled as

$\begin{matrix} k_{\left(h,k\right)}\left(x,y,l\right)=\left(\alpha_{g\left(h,k\right)}+\beta_{g\left(h,k\right)}\sqrt{x}+\delta_{g\left(h,k\right)}l+\gamma_{g\left(h,k\right)}l\sqrt{x}+\varepsilon_{g}\right)^{2}\varsigma_{\Omega\left(h,k\right)} \\ \varepsilon_{g}\sim\text{N}\left(\mu=0,\sigma=\sigma_{g}\right) \end{matrix}$ (3)

| \| Table A2. Analysis of variance of the effects of initial size (S), year (Y), homegarden (H) and proportion of harvested leaves (P) on the growth (final size) of plants with aboveground stems. Sizes were square-rooted before analysis. Only significant terms or those included in higher-order interactions are shown. \| \| \| \| \| \| --- \| --- \| --- \| --- \| --- \| \|  \| Source \| d.f. \| *F* \| *P* \| \| *Sabal yapa* \| S \| 1,217 \| 49497.81 \| <0.001 \| \|  \| Y \| 2,217 \| 5.28 \| 0.006 \| \|  \| S×H \| 1,217 \| 9.99 \| 0.002 \| \| *S. mexicana* \| S \| 1,321 \| 40335.98 \| <0.001 \| \|  \| Y \| 2,321 \| 13.14 \| <0.001 \| \|  \| H \| 1,321 \| 0.44 \| 0.510 \| \|  \| P \| 1,321 \| 2.08 \| 0.151 \| \|  \| S×Y \| 2,321 \| 2.39 \| 0.093 \| \|  \| S×H \| 1,321 \| 0.19 \| 0.662 \| \|  \| Y×H \| 2,321 \| 12.64 \| <0.001 \| \|  \| S×P \| 1,321 \| 0.24 \| 0.625 \| \|  \| Y×P \| 2,321 \| 1.22 \| 0.298 \| \|  \| H×P \| 1,321 \| 5.05 \| 0.025 \| \|  \| S×Y×H \| 2,321 \| 0.35 \| 0.704 \| \|  \| S×Y×P \| 2,321 \| 1.06 \| 0.349 \| \|  \| S×H×P \| 1,321 \| 1.24 \| 0.266 \| \|  \| Y×H×P \| 2,321 \| 1.39 \| 0.252 \| \|  \| S×Y×H×P \| 2,321 \| 8.54 \| <0.001 \| |
| --- | --- | --- | --- | --- | --- | --- | --- | --- | --- | --- | --- | --- | --- | --- | --- | --- | --- | --- | --- | --- | --- | --- | --- | --- | --- | --- | --- | --- | --- | --- | --- | --- | --- | --- | --- | --- | --- | --- | --- | --- | --- | --- | --- | --- | --- | --- | --- | --- | --- | --- | --- | --- | --- | --- | --- | --- | --- | --- | --- | --- | --- | --- | --- | --- | --- | --- | --- | --- | --- | --- | --- | --- | --- | --- | --- | --- | --- | --- | --- | --- | --- | --- | --- | --- | --- | --- | --- | --- | --- | --- | --- | --- | --- | --- | --- | --- | --- | --- | --- | --- |

| Table A3. Analysis of deviance of the effects of year (Y), and homegarden (H) on the survival of plants with aboveground stems. The error distribution was assumed to be binomial and a logit link was used. Only significant terms are shown. | | | | |
| --- | --- | --- | --- | --- |
|  | Source | *χ*^2^ | d.f. | *P* |
| *Sabal yapa* | H | 8.1 | 1 | 0.004 |
|  | Y | 8.3 | 2 | 0.016 |
|  | H×Y | 11.5 | 2 | 0.003 |
| *Sabal mexicana* | H | 5.1 | 1 | 0.023 |
|  | Y | 6.8 | 2 | 0.033 |

1.1.2 Transitions from **Ω** to **D**

Sexual reproduction was the only possible route from the **Ω** to the **D** domain. We observed that reproduction started at 3.5 m. After trying a few functions, it was found that the best fit (based on AIC) to the number of fruits *F*(*x*) produced by a palm of a given size *x,* resulted from using a Michaelis-Menten equation with a gamma error

$F\left(x\right)=\frac{\alpha_{F}+\beta_{F}\left(x-3.5\right)}{x-3.5}$ (4)

The use of *x* - 3.5 precludes plants smaller than 3.5 m from reproducing, as it was observed in the field. Using √*x* instead of *x* resulted in a worse fit. Eq. 4 can be linearized, so we used a generalized linear model to fit it. Only size and homegarden had an effect on the reproduction of both species, whereas harvest and year did not (Table A4).

| Table A4. Analysis of deviance of the effects of initial size (S) and Homegarden (H) on the number of fruits produced by of plants with stems larger than 3.5 m. The error was assumed to follow gamma distribution and a reciprocal link was used. The reciprocal of the plant size was fitted. ^a^Size in this analysis was defined as the stem length-3.5. | | | | |
| --- | --- | --- | --- | --- |
|  | Source | *F* | d.f. | *P* |
| *Sabal yapa* | S^a^ | 6.5 | 1,36 | 0.057 |
|  | H | 19.9 | 1,36 | 0.002 |
| *Sabal mexicana* | S^a^ | 3.4 | 1,99 | 0.049 |
|  | H | 8.0 | 1,99 | 0.003 |
|  | S^a^×H | 43.7 | 1,99 | <0.001 |

We assumed that there was no seed bank (palms in general do not form a seed bank (Vazquez-Yanes and Orozco-Segovia (1993); Svenning (2001)), and thus that the number of seedlings observed in any year should come from the fruits produced a few months before. We observed that newly-recruited seedlings could be either in category S1 or S2 by the end of the year. To estimate *φ*, probability that the seed in a fruit becomes established (*i.e.*, that it germinates and survives to the end of each sampling year), we calculated the ratio of the number of fruits produced in the whole homegarden to the number of seedlings estimated for the same area. Because of the estimation errors involved in these figures, it was not possible to correctly calculate *P*-values for the differences in *φ* between years and homegardens and we used the observed values for each year × homegarden combination. Using a log-linear model, we found that the proportion of S1 to S2 seedlings (*θ*) differed across homegardens and years in *S. yapa* (Table A5), but no significant differences were found for *S. mexicana*.

| Table A5. Deviance analysis for the effects of year (Y) and homegarden (H) in the proportion of *S. yapa* S1 to S2 seedlings. The interactions involving seedling type (T) indicate that the proportion is affected by the respective variable. | | |
| --- | --- | --- |
| Source | *χ*^2^ | *P* |
| H | 30.93 | <0.001 |
| Y | 67.67 | <0.001 |
| T | 6.65 | 0.010 |
| H×Y | 59.09 | <0.001 |
| H×T | 0.05 | 0.826 |
| Y×T | 0.09 | 0.759 |
| H×Y×T | 9.56 | 0.002 |

Thus, the number of S1 and S2 seedlings produced by a size *x* mother plant (respectively *f*_S1_(*x*) and *f*_S2_(*x*)) was estimated as

 (5)

1.1.3. Transitions within the **D** domain

We modeled the behavior of plants without stem as the multiplication of the growth and survival 5×5 matrices

**A**=**GS** (6)

where the *γ_i,j_* elements of **G** correspond to the probability that an individual in category *j* transits to category *i* given that it survives**,** and the elements of the diagonal matrix **S,** *ς_j_*, correspond to the survival probability of individuals in the category *j*. To determine which factors affect *ς_i_*, a model was fitted using homegarden, year and category as explanatory variables, and a binary response variable consisting of 0s and 1s depending on whether a given individual survived after one year. We assumed a binomial error. Survival probability was significantly affected by the interaction of year, homegarden and initial stage in both species (Table A6), indicating that the use of different survival probabilities for each condition is justified. A separate analysis was then conducted using only the individuals in category I3 in order to assess if there were differences in survival depending on harvest intensity, but no effect was found.

| Table A6. Analysis of deviance of the effects of year (Y), initial stage (IS) and homegarden (H) on plant survival. The error distribution was assumed to be binomial and a logit link was used. | | | | |
| --- | --- | --- | --- | --- |
|  | Source | *χ*^2^ | d.f. | *P* |
| *Sabal yapa* | Y | 71.7 | 2 | <0.001 |
|  | IS | 239.4 | 4 | <0.001 |
|  | H | 0.7 | 1 | 0.389 |
|  | Y×IS | 6.6 | 8 | 0.583 |
|  | Y×H | 10.2 | 2 | 0.006 |
|  | IS×H | 14.6 | 4 | 0.006 |
|  | Y×IS×H | 22.5 | 8 | 0.004 |
|  |  |  |  |  |
| *Sabal mexicana* | Y | 12.1 | 2 | 0.002 |
|  | IS | 357.6 | 4 | <0.001 |
|  | H | 60.1 | 1 | <0.001 |
|  | Y×IS | 3.5 | 8 | 0.901 |
|  | Y×H | 6.3 | 2 | 0.042 |
|  | IS×H | 8.9 | 4 | 0.063 |
|  | Y×IS×H | 16.9 | 8 | 0.031 |

We found significant effects of year and homegarden on growth by means of a log-linear model using these variables and the category in which individuals where at time *t* and *t* +1as explanatory variables, and the number of individuals observed in each combination of factors as a response variable. In such models, significant interactions between the category to which individuals transit and an environmental variable indicate that the latter had an effect on growth (Caswell (2001)). We assumed a Poisson error for *S. yapa.* In the case of *S. mexicana* we found overdisperssion, so a quasipoisson error was used for that model. The analyses were then repeated for each category separately to identify which ones were significantly affected by the differences between years and homegardens. In the models for I3 individuals, harvest intensity was also included. A sixth category representing the transition to the **Ω** domain was included in the models for I3 plants. Growth of individuals did not differ significantly among years or homegardens except for I2 and I1 individuals in *S. yapa* and *S mexicana,* respectively. No effect of leaf harvesting was found (Table A7).

| Table A7. Analysis of deviance of the effects of year (Y) and homegarden (H) on the number of individuals transiting to a given stage (FS) depending on the initial stage and species. The error distribution was assumed to be ^a^Poisson or ^b^Quasi-Poisson, and a log link was used. | | | | |
| --- | --- | --- | --- | --- |
|  | Source | *χ*^2^ | d.f. | *P* |
| *S. yapa* I2^a^ | Y | 5.9 | 2 | 0.051 |
|  | H | 6.0 | 1 | 0.014 |
|  | FS | 167.1 | 4 | <0.001 |
|  | H×FS | 14.1 | 4 | 0.007 |
|  |  |  |  |  |
| *S.mexicana* I1^b^ | Y | 19.5 | 2 | <0.001 |
|  | H | 21.2 | 1 | <0.001 |
|  | FS | 128.8 | 5 | <0.001 |
|  | Y×FS | 6.9 | 2 | 0.031 |
|  | H×FS | 11.2 | 4 | 0.024 |

Thus, transitions within the **D** domain were modeled with the matrix (zeroes in the **G** matrix correspond to transitions that were never observed):

$A=\left[\begin{matrix} \gamma_{\text{S1,S1}} & 0 & 0 & 0 & 0 \\ \gamma_{\text{S2,S1}} & \gamma_{\text{S2,S2}} & 0 & 0 & 0 \\ \gamma_{\text{I1,S1}} & \gamma_{\text{I1,S2}} & \gamma_{\text{I1,I1}\left(h,k\right)} & 0 & 0 \\ 0 & \gamma_{\text{I2,S2}} & \gamma_{\text{I2,I1}\left(h,k\right)} & \gamma_{\text{I2,I2}\left(h\right)} & \gamma_{\text{I2,I3}} \\ 0 & 0 & \gamma_{\text{I3,I1}\left(h,k\right)} & \gamma_{\text{I3,I2}\left(h\right)} & \gamma_{\text{I3,I3}} \end{matrix}\right]\left[\begin{matrix} \varsigma_{\mathrm{S}1\left(h,k\right)} & 0 & 0 & 0 & 0 \\ 0 & \varsigma_{\mathrm{S}2\left(h,k\right)} & 0 & 0 & 0 \\ 0 & 0 & \varsigma_{\text{I1}\left(h,k\right)} & 0 & 0 \\ 0 & 0 & 0 & \varsigma_{\text{I2}\left(h,k\right)} & 0 \\ 0 & 0 & 0 & 0 & \varsigma_{\text{I3}\left(h,k\right)} \end{matrix}\right]$(7)

The values of *ς_j_* and *γ_i,j_* were estimated as the ratio of the number of individuals that survived or reached category *i*, respectively, to the number of individuals in category *j.* When no significant differences were found between years or homegardens, we used the pooled dataset to estimate *γ_i,j_*.

1.1.4. Transitions from **D** to **Ω**

Only individuals in the category I3 developed stems over one annual transition. This transition from the **D** to the **Ω** domain requires a probability density function for the sizes of individuals that develop a stem for the first time. We assumed normality for the square-rooted final size because growth was found to follow this distribution. We tested whether year, harvest, and homegarden had an effect on the mean size of newly produced stems using an ANOVA. We found no significant effects of any variable, probably due the small number of individuals that made this transition successfully and thus a lack of statistical power. Thus, we modeled the probability of reaching a size *y* as

$\sqrt{y}\sim\text{N}\left(\mu_{y},\sigma_{y}\right)$ (8)

For an I3 individual to reach such size, it must survive and develop a stem, so we multiplied the probability density in (8) by *ς*_I3(_*_h,k_*_)_ and *γ***_Ω_**_,I3_, the probability that a plant develops a stem. The latter did not differ between years or homegardens, nor with harvest, as per the results in the previous section.

*1.2. Observed patterns in vital rates*

All the parameters of the GIMP are summarized in table A8. The following sections highlight some of the patterns found in vital rates that are relevant for the discussion of the results, but are not presented in the main text.

Table A8. Parameters of the generalized integral proyection model for two *Sabal* species in two homegardens in three different years: 1998, 1999 and 2000. Se table A1 for parameter meanings.

|  | *Sabal yapa* | | | | | | *Sabal mexicana* | | | | | |
| --- | --- | --- | --- | --- | --- | --- | --- | --- | --- | --- | --- | --- |
| Para-meter | Homegarden 1 | | | Homegarden 2 | | | Homegarden 1 | | | Homegarden 2 | | |
|  | 98 | 99 | 00 | 98 | 99 | 00 | 98 | 99 | 00 | 98 | 99 | 00 |
| *α_F_* | 0.00035 | 0.00035 | 0.00035 | 0.00002 | 0.00002 | 0.00002 | 0.00006 | 0.00006 | 0.00006 | 0.00027 | 0.00027 | 0.00027 |
| *α_g_* | 0.2785 | 0.2746 | 0.0807 | 0.2933 | 0.2894 | 0.0955 | 0.1821 | 0.5374 | 0.9260 | 1.2414 | -0.4980 | -0.4871 |
| *β_F_* | 0.0073 | 0.0073 | 0.0073 | 0.0073 | 0.0073 | 0.0073 | 0.1489 | 0.1489 | 0.1489 | 0.0006 | 0.0006 | 0.0006 |
| *β_g_* | 1.0026 | 1.0026 | 1.0026 | 1.0026 | 1.0026 | 1.0026 | 1.0234 | 0.9867 | 0.9506 | 0.9351 | 1.0648 | 1.0560 |
| *γ_g_* | 0 | 0 | 0 | 0 | 0 | 0 | 0.1372 | -0.8162 | -0.9194 | -1.1716 | 2.5382 | 1.0463 |
| *δ_g_* | 0 | 0 | 0 | 0 | 0 | 0 | -0.0362 | 0.9627 | 0.9932 | 0.1281 | -0.1761 | -0.1001 |
| *γ*_S1,S1_ | 0 | 0 | 0 | 0 | 0 | 0 | 0.0455 | 0.0455 | 0.0455 | 0.0455 | 0.0455 | 0.0455 |
| *γ*_S2,S1_ | 0.9231 | 0.9231 | 0.9231 | 0.9231 | 0.9231 | 0.9231 | 0.9545 | 0.9545 | 0.9545 | 0.9545 | 0.9545 | 0.9545 |
| *γ*_I1,S1_ | 0.0769 | 0.0769 | 0.0769 | 0.0769 | 0.0769 | 0.0769 | 0 | 0 | 0 | 0 | 0 | 0 |
| *γ*_S2,S2_ | 0.7952 | 0.7952 | 0.7952 | 0.7952 | 0.7952 | 0.7952 | 0.9462 | 0.9462 | 0.9462 | 0.9462 | 0.9462 | 0.9462 |
| *γ*_I1,S2_ | 0.1747 | 0.1747 | 0.1747 | 0.1747 | 0.1747 | 0.1747 | 0.0538 | 0.0538 | 0.0538 | 0.0538 | 0.0538 | 0.0538 |
| *γ*_I2,S2_ | 0.0301 | 0.0301 | 0.0301 | 0.0301 | 0.0301 | 0.0301 | 0 | 0 | 0 | 0 | 0 | 0 |
| *γ*_I1,I1_ | 0.7900 | 0.7900 | 0.7900 | 0.7900 | 0.7900 | 0.7900 | 0.8519 | 0.3500 | 0.7143 | 0.2857 | 0.2857 | 0.2857 |
| *γ*_I2,I1_ | 0.1900 | 0.1900 | 0.1900 | 0.1900 | 0.1900 | 0.1900 | 0.1481 | 0.6500 | 0.2857 | 0.6429 | 0.6429 | 0.6429 |
| *γ*_I3,I1_ | 0.0200 | 0.0200 | 0.0200 | 0.0200 | 0.0200 | 0.0200 | 0 | 0 | 0 | 0.0714 | 0.0714 | 0.0714 |
| *γ*_I2,I2_ | 0.8704 | 0.8704 | 0.8704 | 0.5135 | 0.5135 | 0.5135 | 0.6392 | 0.6392 | 0.6392 | 0.6392 | 0.6392 | 0.6392 |
| *γ*_I3,I2_ | 0.1296 | 0.1296 | 0.1296 | 0.4865 | 0.4865 | 0.4865 | 0.3608 | 0.3608 | 0.3608 | 0.3608 | 0.3608 | 0.3608 |
| *γ*_I2,I3_ | 0 | 0 | 0 | 0 | 0 | 0 | 0.0094 | 0.0094 | 0.0094 | 0.0094 | 0.0094 | 0.0094 |
| *γ*_I3,I3_ | 0.9552 | 0.9552 | 0.9552 | 0.9552 | 0.9552 | 0.9552 | 0.8396 | 0.8396 | 0.8396 | 0.8396 | 0.8396 | 0.8396 |
| *γ*_Ω,I3_ | 0.0448 | 0.0448 | 0.0448 | 0.0448 | 0.0448 | 0.0448 | 0.1509 | 0.1509 | 0.1509 | 0.1509 | 0.1509 | 0.1509 |
| *θ* | 0.3720 | 0.4513 | 0.2927 | 0.3382 | 0.3431 | 0.3333 | 0.3786 | 0.3786 | 0.3786 | 0.3786 | 0.3786 | 0.3786 |
| *μ_y_* | 3.5519 | 3.5519 | 3.5519 | 3.5519 | 3.5519 | 3.5519 | 3.0711 | 3.0711 | 3.0711 | 3.0711 | 3.0711 | 3.0711 |
| *σ_g_* | 0.4150 | 0.4150 | 0.4150 | 0.4150 | 0.4150 | 0.4150 | 0.4749 | 0.4749 | 0.4749 | 0.4749 | 0.4749 | 0.4749 |
| *σ_y_* | 0.8512 | 0.8512 | 0.8512 | 0.8512 | 0.8512 | 0.8512 | 0.5220 | 0.5220 | 0.5220 | 0.5220 | 0.5220 | 0.5220 |
| *ς*_1_ | 0.3333 | 0.7500 | 0 | 0.3333 | 0 | 0.1333 | 0.4000 | 0.2500 | 0.0571 | 0.0417 | 0 | 0.2500 |
| *ς*_2_ | 0.6708 | 0.7297 | 0.2232 | 0.6363 | 0.5167 | 0.6071 | 0.6938 | 0.4949 | 0.2992 | 0.0625 | 0.1290 | 0.1000 |
| *ς*_3_ | 0.9473 | 0.8947 | 0.8889 | 0.9091 | 0.9167 | 0.8571 | 0.9000 | 0.8696 | 0.7778 | 0.8571 | 0.2500 | 1.0000 |
| *ς*_4_ | 0.8695 | 0.9444 | 0.8947 | 0.8000 | 0.8235 | 0.5384 | 0.9412 | 0.9333 | 1.0000 | 0.9333 | 0.9130 | 0.7500 |
| *ς*_5_ | 0.9561 | 0.9545 | 1.0000 | 0.9523 | 0.9629 | 0.7667 | 1.0000 | 0.9600 | 0.9629 | 1.0000 | 1.0000 | 1.0000 |
| *ς*_Ω_ | 0.9722 | 0.7297 | 1.0000 | 0.9783 | 1.0000 | 0.9583 | 0.9737 | 0.9627 | 0.9932 | 0.9267 | 0.8981 | 0.9802 |
| *φ* | 0.0243 | 0.0112 | 0.0013 | 0.0096 | 0.0003 | 0.0037 | 0.0157 | 0.0191 | 0.0016 | 0.0002 | 0.0008 | 0.0010 |

1.2.1. **Ω** domain

Compared with H2, survival of *S. mexicana* was greater in H1, but lower for *S. yapa*. In general, survival was lowest in the second year, except for *S. yapa* in H1 (Figure A1). Fecundity was always greater in larger individuals and in H2 (Figure A2)

Figure A1. Survival probabilities of stemmed palms of both species in each homegarden averaged across years.

Figure A2: Number of fruits produced by *Sabal yapa* (upper panel) and *S. mexicana* (lower panel) depending on stem size. Results for homegarden 1 are shown in black, and for homegarden 2 in gray

**D** *domain*

The survival probability increased monotonically as the plants developed, changing from 0.15 in S1 to 0.95 in I3 plants. On average, survival was larger for *S. yapa* in H1 (0.58, compared to 0.68 in H2), but the opposite occurred in *S. mexicana* (0.56 in H1, 0.43 H2; Table A8). No simple pattern was found for growth (Table A8)

**2. Harvest model**

*2.1 Estimation of the number of leaves available for harvest*

As explained in the main text, we used the following equation to estimate the number of leaves available in a palm

$L_{t+1}=\min(L_{t}-L_{h,t}+L_{p,t},L_{\max})$, (9)

where *L_t_* is the number of leaves available in an individual at time *t*, *L_h,t_*_-1_ is the previous leaf harvest, the number of leaves produced is *L_p,t_*_-1_, and the maximum number of leaves that a palm may have is *L*_max_ that an individual may have.

To estimate *L*_max_ and to assess if this number depends on palm size, we conducted a stochastic frontier analysis using the package frontier (Coelli and Henningsen (2013)) for R. This regression procedure does not estimate the mean value of the response variable, but its maximum instead. We found that the model fit was best if we regressed the log of the number of leaves in a palm on the square root of its height. The gamma parameter of the regressions were significant (*S. yapa*: *γ* = 0.8114, *P* <0.0001, *S. mexicana*: *γ* = 0.7972, *P* <0.0001), indicating that the use of a stochastic frontier model is preferable over an ordinary least-squares regression, and that there was in fact a maximum number of leaves that a palm may have (Coelli (1994)). For both species, *L*_max_ increased with size (*P* < 0.0001) from about 7 leaves in smaller individuals to slightly over 20 in the largest ones. The equations used to estimate *L*_max_ for *S. yapa* and *S. mexicana* were, respectively,

$\begin{matrix} L_{\max}=e^{\text{2.045+0.0375}\sqrt{x}} \\ L_{\max}=e^{\text{2.134+0.0300}\sqrt{x}} \end{matrix}$. (10)

We modeled the production of new leaves *L_p,t_* as a function of stem size, homegarden, year and harvest through generalized additive models (GAMs). We assumed a Poisson error and a log link using package mgcv (Wood (2011)) for R. Because leaf production may depend on leaf harvest in the previous year, we conducted an analysis using only years 2 and 3 for which leaf harvest in the previous year was known. Harvest on one year had no effect on leaf production during the following year (*P* = 0.0732 and *P* = 0.8154 for *S. yapa* and *S. mexicana*, respectively), so we included only harvest at time *t* to model *L_p,t_*. We tried the proportion or the number of leaves harvested as measures of harvest intensity, and found that the latter resulted in a lower AIC value. For both species, leaf production depended on the number of leaves harvested, plant size, homegarden and year (Table A9).

| Table A9. Analysis of deviance of the effects of year (Y), homegarden (H), size (S), and number of leaves harvested (N) on the number of leaves produced. The error distribution was assumed to be Poisson and a log link was used. | | | | |
| --- | --- | --- | --- | --- |
|  | Source | *χ*^2^ | d.f. | *P* |
| *S. yapa* | H | 6.14 | 0.21 | 0.002 |
|  | N | 24.89 | 1.48 | <0.001 |
|  | S | 33.24 | 3.33 | <0.001 |
|  | Y | 6.24 | 1.25 | 0.012 |
|  | H×N | 8.82 | 1.94 | 0.011 |
|  | H×Y | 11.23 | 2.40 | 0.006 |
|  | S×Y | 44.10 | 7.33 | <0.001 |
|  | S×H | 13.88 | 4.48 | 0.012 |
|  | S×H×Y | 43.20 | 8.22 | <0.001 |
|  | H×Y×N | 12.57 | 3.67 | 0.010 |
|  |  |  |  |  |
| *S.mexicana* | Y | 308.01 | 0.45 | <0.001 |
|  | N | 750.18 | 1.52 | <0.001 |
|  | S | 176.00 | 1.53 | <0.001 |
|  | S×H | 114.60 | 5.72 | 0.002 |

*2.2 Observed harvest intensity*

For the simulations with the observed harvest intensity we need a function relating size and harvest. We obtained it through GAMs using the proportion of leaves harvested assuming a quasibinomial error (due to overdispersion) and a logit link. In *S. mexicana*, homegarden, year, and size had a significant effect on harvest, whereas in *S. yapa* no differences between years were detected (Table A10). The fitted models show a very complex behavior, but confirm that Maya do not harvest palms above 3 m tall (Figure A3)

| Table A10. Analysis of deviance of the effects of year (Y), homegarden (H) and size (S) on the fraction of leaves harvested. The error distribution was assumed to be quasibinomial and a logit link was used. | | | | |
| --- | --- | --- | --- | --- |
|  | Source | *χ*^2^ | d.f. | *P* |
| *S. yapa* | H | 33.21 | 0.89 | <0.001 |
|  | S | 316.31 | 3.81 | <0.001 |
|  | S×H | 33.37 | 4.00 | <0.001 |
|  |  |  |  |  |
| *S.mexicana* | H | 11.89 | 1.76 | 0.002 |
|  | Y | 11.49 | 1.58 | 0.002 |
|  | S | 535.06 | 2.77 | <0.001 |
|  | H×Y | 35.06 | 0.92 | <0.001 |
|  | S×H | 54.56 | 6.99 | <0.001 |
|  | S×H×Y | 123.58 | 14.59 | <0.001 |

Figure A3. Observed harvest intensities for *S. yapa* (Upper panel) and S. *mexicana* (Lower panel). Black: homegarden 1; grey: homegarden 2. Solid: 1998; dashed: 1999; dotted: 2000.

By means of a generalized linear model with a quasibinomial error, we found that, for I3 individuals, the fraction of leaves harvested differed significantly between years and homegardens is *S. yapa* (homegardens *χ*^2^=4.2535 d.f. = 1, *P* = 0.041, years *χ*^2^=43.31 d.f. = 2, , *P* <0.001). In *S. mexicana*, harvest of I3 plants was constant.

**3. Simulations**

The dynamics of the population was simulated from eqn. (1). Numerical integration was done by partitioning the **D** domain into 200 categories. Because growth of stemmed palms was a function of the square-rooted stem length, the partition was done on the square-rooted size. This has the advantage of using a narrower partition for small plants where vital rates change more rapidly with size, and thus may render more accurate results.Temporal variability was introduced in the model by means of a stochastic GIPM (Ellner and Rees (2007)) in which years were chosen randomly each iteration. Because in *S. yapa* the vital rates were unaffected by harvest, the same three annual kernels were used over the simulation. This means that the population growth rate and size structure were the same regardless of harvest. However, in *S. mexicana* the kernel changes with the proportion of harvested leaves. In those simulations in which people are assumed to leave a constant number of leaves in the palm and harvest the rest, the proportion of harvested leaves changes every year, so a new kernel had to be estimated in each iteration. See main text for further details.

**References**

Coelli TJ and Henningsen A (2013) frontier: Stochastic Frontier Analysis. R package version 1.0. <http://CRAN.R-Project.org/package=frontier>.

Coelli TJ (1994) A guide to frontier 4.1: A computer programme for stochastic frontier production and cost function estimation, NSW 2351 Armidale, Australia: Department of Econometrics, University of New England.

Caswell H (2001) Matrix population models: construction, analysis, and interpretation. Sinauer Associates, Inc., Sunderland, Massachusetts. 2nd:722.

Ellner SP, Rees M (2006) Integral projection models for species with complex demography. Am Nat 167:410–428.

Ellner SP, Rees M (2007) Stochastic stable population growth in integral projection models: Theory and application. J Math Biol 54:227–256.

Svenning JC (2001) On the Role of Microenvironmental Heterogeneity in the Ecology and Diversification of Neotropical Rain-Forest Palms (Arecaceae). Bot Rev 67:1–53.

Vazquez-Yanes C, Orozco-Segovia A (1993) Patterns of seed longevity and germination in the tropical rainforest. Annu Rev Ecol Syst 24:69–87.

Wood SN (2011) Fast stable restricted maximum likelihood and marginal likelihood estimation of semiparametric generalized linear models. J R Stat Soc Ser B (Statistical Methodol 73:3–36. doi: 10.1111/j.1467-9868.2010.00749.x
